# Ant trophallactic networks: simultaneous measurement of interaction patterns and food dissemination

**DOI:** 10.1038/srep12496

**Published:** 2015-07-30

**Authors:** Efrat Greenwald, Enrico Segre, Ofer Feinerman

**Affiliations:** 1 Department of Physics of Complex Systems, Weizmann Institute of Science, Rehovot, Israel; 2 Department of Physics Services Unit, Weizmann Institute of Science, Rehovot, Israel

## Abstract

Eusocial societies and ants, in particular, maintain tight nutritional regulation at both individual and collective levels. The mechanisms that underlie this control are far from trivial since, in these distributed systems, information about the global supply and demand is not available to any single individual. Here we present a novel technique for non-intervening frequent measurement of the food load of all individuals in an ant colony, including during trophallactic events in which food is transferred by mouth-to-mouth feeding. Ants are imaged using a dual camera setup that produces both barcode-based identification and fluorescence measurement of labeled food. This system provides detailed measurements that enable one to quantitatively study the adaptive food distribution network. To demonstrate the capabilities of our method, we present sample observations that were unattainable using previous techniques, and could provide insight into the mechanisms underlying food exchange.

Networks occur in a multitude of systems whether they are physical, biological, or man-made^1^,^2^,^3^,^4^. Whereas a theoretical understanding of network structure is highly developed, network function and dynamics are still poorly understood^5^. The main reason for this is the lack of empirical datasets that contain all relevant aspects: namely, network structure, dynamics, and function^6^.

Social insect colonies have key network attributes and hence serve as a live model for studying dynamical networks^7^,^8^,^9^. Interactions between insect workers define the connectivity of these networks, which is nonrandom and evolves with time^10^. These biological networks are of special interest since they accommodate highly robust and tightly regulated collective behaviors^11^,^12^,^13^,^14^.

A striking example of a tightly controlled network function involves food sharing, a fundamental behavior in eusocial insects^15^,^16^,^17^,^18^,^19^. Generally, only a small fraction of workers is responsible for collecting food, which is later distributed to the entire population of the colony^20^,^21^. It was demonstrated that ant and bee colonies tightly regulate all aspects of this process including the global nutritional intake^22^,^23^,^24^ and the dissemination of food to different subpopulations, each of which has different nutritional needs^20^,^25^,^26^,^27^. Nutrition intake is regulated not only at the population level but also by each individual within the colony^28^. Interestingly, this multi-scale process emerges from the interactions between individuals and not from some form of central control.

A central mechanism underlying food dissemination in social insects is trophallaxis, which is mouth-to-mouth transfer of liquid food between individuals^16^,^17^,^29^. Ants and bees can store a considerable amount of liquids in their crops, a pre-digestion storage organ^17^,^30^,^31^. During a trophallactic interaction, food stored in one worker’s crop is regurgitated and passed on to the other. In light of this process, the crop is often referred to as a “social stomach”^32^. Since trophallaxis involves physical interactions between workers, it constitutes a temporal interaction network^33^,^34^.

Previous techniques for measuring the crop liquids of ants and bees often focused either on the individual level or on that of the entire colony. Methods that relate to the scale of single ants typically use food that is marked by dyes^35^,^36^, radioactive markers^15^,^20^,^37^,^38^,^39^ or fluorescent beads^40^ and that is made available to the insects for a given duration. Thereafter, crop liquid contents are measured by inspecting single insects using a microscope, a radioactive counter, or scales. Although these methods yield accurate individual measurements, they are limited by their intrusiveness and, to some extent, by their sample size. Colony-level measurements were previously obtained by scintigraphy, a radioactivity based medical imaging technique^41^. This undisruptive method provides repeated measurements of food spread dynamics within the colony. Complementing this technique with webcam imagery established the spatial^39^ correlations between food accumulation and ant distribution within the nest. The low spatiotemporal resolution obtained by this method is its main shortcoming: The temporal resolution is 30 seconds, which is longer than the majority of trophallactic events, whereas the spatial resolution does not allow for individual-based measurements. A method that addresses both individual and collective scale processes uses video imaging to manually identify trophallactic events of individually color-painted ants^34^. The direction of the food flow was determined by mandible postures, whereas the duration of the trophallactic events was used as a proxy for the amount of food transferred. We discuss the accuracy of this video-based method below.

In the present work we introduce a novel technique for tracking the dissemination of food within an ant colony by combining the imaging of fluorescently labeled food with the video tracking of ant trajectories using 2D barcode identification. A dual camera setup enables frequent measurement of liquid food within the crops of all individual ants in the colony, with a spatiotemporal resolution that allows one to observe single trophallactic events, without interfering with the colony (Fig. 1). In the results section, we show a linear relation between the measured fluorescence levels and the actual crop liquid contents. We then estimate the accuracy of this relation to quantify the capabilities and limitations of our technique. We go on to describe several experiments that serve as sample cases and are aimed at demonstrating the opportunities made available by this new system. To summarize, our method enables the exploration of the process of nutritional regulation by both the individual and the colony and provides a unique opportunity to study a natural adaptive network together with the resources that flow over it.

## Materials and Methods

### Ant species

The system was tested using two different ant species: *Camponotus sanctus* and *Campontus fellah*. These species are characterized by relatively large (1–2 cm) workers that have a partially translucent gaster. These properties make them suitable for both barcode labeling and crop imaging techniques. Our measurements focus on lab colonies reared from single queens that were collected during nuptial flights in the Neve Shalom and Rehovot areas in Israel.

### Food sources

Ants were fed with four different water-based food solutions: Sucrose (C_12_H_22_O_11_) and glucose (C_6_H_12_O_6_) solutions were used as carbohydrate sources, Bovine Serum Albumin (BSA) and Adenylate Kinase solutions as protein sources.

Fluorescent markers were added to water-based solutions of sucrose, glucose and BSA by dissolving Rhodamine B (C_28_H_31_ClN_2_O_3_, λ_ex_ 553 nm, λ_em_ 627 nm) at a concentration of 0.08 mg/ml (unless otherwise specified) into the liquid solution. This concentration was chosen as the maximal concentration for which fluorescence levels linearly scale with liquid volume as measured within microwells. This scaling persists up to a depth of 5 mm, which is larger than the typical thickness of an ant’s gaster (see the calibration in Fig. 2). Rhodamine B is not toxic to the ants even at high concentration of 10 g/l. Colonies that had the high concentration dyed solution as an exclusive food source for several months exhibit normal death rates throughout this period.

We further used a protein source in which the molecules are directly fluorescently tagged: Adenylate Kinase labeled at position 169 with the fluorescent marker ATTO590 (kindly supplied by the Gilad Haran group at the Weizmann Institute of Science).

### Nutrient transfer

We use the terms crop contents or crop liquid content to describe our measurements. When the fluorescent marker is bound to the nutrient itself, like in the case of the protein Adenylate Kinase, fluorescence measurements are directly indicative of the protein content (see SI-1). In practice, most of the experiments presented here measure non-bound fluorescent markers transferred within the solution. In the case of the Sucrose Rhodamine B solution, since the molecules of Rhodamine B and sucrose are of, roughly, the same size and both are water-soluble it is reasonable to expect that they are both transferred together during trophallaxis. This suggests that fluorescence measurements are a good proxy for sucrose content (this assumption has been the basis of multiple studies^27^,^35^,^37^).

We conducted control experiments to test whether ants may filter the contents of their crop during trophallaxis such that they transfer the fluorescent dye but not the actual sugar. To do so we compared ants that were directly fed by a glucose and Rhodamine B solution to ants that were fed via trophallaxis alone. Our results show that the glucose concentration of the crop liquids of ants fed solely by trophallaxis was similar to that of ants that had been directly fed, and much above the concentrations measured for the starved ants. These measurements demonstrate that, during trophallaxis, the ants indeed transfer glucose rather than dye molecules alone. For a more detailed description of this experiment refer to SI-1.

The impact of fluorescence decay due to digestion was tested by measuring the fluorescence levels of isolated workers that have been fed by a fluorescent glucose solution. The fluorescence signal decreased by only 10% over 15 hours (see SI-7), whereas food dissemination is essentially over in less than an hour. This long time scale is also consistent with the previous measurements of the flow of food from the crop to the stomach^40^. This implies that fluorescence can be reliably measured for at least several hours before massive digestion occurs.

### The experimental setup

**Figure 1:**
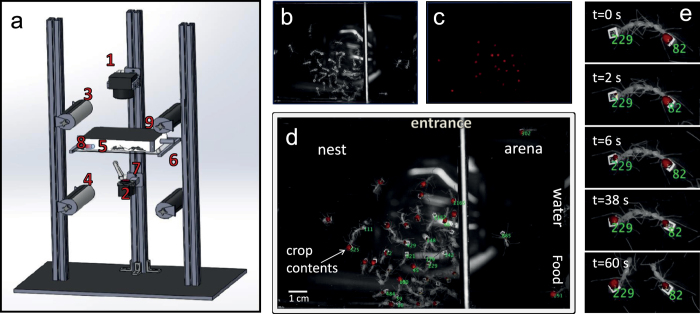
The experimental system. (**a**) Illustration of the setup: 1. Upper camera 2. Lower camera. 3. IR illumination 4. Fluorescent illumination. 5. Artificial nest 6. Transparent plate. 7. Fluorescence filter. 8. Fluorescently labeled food 9. IR filter. (**b**) Upper camera image of tagged ants in the experimental arena. (**c**) Lower camera image of fluorescently labeled food within individual crops. (**d**) An image combining images b and c that depicts both the ants’ identities (green labels) and their crop contents (red blobs). (**e**) From top to bottom: time sequence images of a trophallactic event, in which liquid (red blobs) passes from ant 82 to ant 229.

Ants are imaged using two cameras that are aligned above and below^42^ the experimental arena (Fig. 1a). The upper camera (Vieworks 25 M) is used to identify the ants by tracking the barcodes attached to their dorsal side (Fig. 1b). The fluorescence camera (Prosilica GC2450) is placed below and images the ants’ crops through a transparent floor (Fig. 1c). The fluorescence camera serves as a master camera: it triggers both the upper camera and the fluorescent illumination so that the images obtained by both cameras are synchronized. Image management and control of camera attributes are done using the LabVIEW interface.

Infrared lamps (850 nm) are used as a light source for the upper camera. The nest chamber is covered with an infrared filter which makes the inside of the nest appear dark to the ants that have no IR vision^43^ (apart from the brief periods of green pulses, see below). A small, uncovered area outside the nest functions as a yard, where labeled food and water are supplied to the ants.

Fluorescent illumination is provided by millisecond pulses of high-power green LEDs (principal wavelength: 530 nm, LUXEON Rebel LED - 161 lm @ 700 mA). The experimental nest area (~100 cm^2) is uniformly illuminated by 30 LEDs, located beneath the arena. Pulses were administered at 0.5 Hz and their duration was as brief as possible, in order to diminish light disruptions to the ants (see SI-2), as well as to reduce bleaching which we measure to be negligible on a time scale of five hours (see SI-3). The emitted fluorescent light is passed through a red filter (Lee filter 106) and collected by the lower camera.

### Barcode technology and image analysis

Ants are tagged with 1 mm^2^ stamps (printed at 2540 DPI on a “Dolev 800” printer) containing 36 bit (6 × 6) 2D barcodes (BugTag, Robiotec), which are gently attached to the ants’ gasters (either to the postpetiole or to the tergite, but not in between, Fig. 1b) using a small drop of skin adhesive^33^ (SAUR-HAUTKLEBER 50.20-2% Harz). Individual ants are identified by a commercial computer vision-based tracking system (BugTag, Robiotec) that enables recognition of up to several hundred individual ants. To reliably decode the tags, each tag has to occupy 784 (28 × 28) pixels on the image frame. Taken together with the total number of pixels per image (*i.e*. camera resolution), this constraint dictates a maximal size for the experimental arena (see SI- 4). Images are analyzed with the Matlab Image Processing Toolbox. Briefly, the fluorescence images are analyzed by subtracting a background image and identifying all pixels whose intensity crosses a certain threshold that is set just above the ants’ auto-fluorescence level. These bright pixels are then grouped into connected components (blobs) that correspond to the gasters of single ants. The total intensity of each blob is defined as the sum of the intensities of all the pixels it includes. Images collected by the upper camera are analyzed by the tracking software to obtain the locations and orientations of uniquely identified ants. Using the Munkres algorithm^44^, each identified ant is then assigned a fluorescence blob that corresponds to the content of her gaster, according to spatial proximity (see SI-6). Following this analysis, a timeline for each identified ant can be produced, including her spatial position and crop liquid content.

In order to increase the accuracy of our data, trophallactic events are identified manually from the video generated by the upper camera. Interactions are classified as trophallactic events whenever the mandibles of the participating workers touch each other and at least one of the mandibles is open. The crop liquid content of the participating ants is then extracted for the entire course of the event (see Fig. 3a).

### Calibration of the fluorescence signal

A calibration curve was constructed from individual ant measurements. As they were feeding (a water-based solution of sucrose (80 g/l) and Rhodamine B (0.08 mg/ml)), the ants were interrupted several times to be weighed and fluorescently imaged. Each of the seven measured individuals included 4–8 measurements, which in total summed up to 31 samples. One can consider these as independent samples each holding the amount of extra food an ant ate and the increment in her fluorescence signal. All fluorescence measurements included a series of at least ten images such that, at least in some of them, all six of the ant’s legs touch the floor. The images were analyzed as described in the previous section, and the maximal measurement was chosen as the best estimation. The uncertainty in the food mass evaluation given a specific fluorescence measurement, was estimated by following Miller’s approximation^45^ for confidence intervals (for details, see SI-5).

Since ant posture is the main source of error in our experimental measurements (see the Calibration and Error Estimation section) and assuming that the ants’ crop liquid content remains constant between trophallactic events, the maximal fluorescence measurement acquired during each interval between events is considered as the most reliable evaluation of the crop liquid content. This evaluation of crop liquid content improves as the time interval between consecutive trophallactic events increases and more fluorescence measurements are obtained. To assess how measurement errors decrease with time (given that no additional trophallaxis event has occurred) we focused on time intervals that were longer than 70 seconds (N = 350 intervals) so that acquiring at least one good quality image of the ant (no obstructed body parts) is highly probable. For each time t within such an interval, the ratio between the maximal fluorescence measurement obtained by time t and the global maximal measurement over the entire interval was calculated. The improvement in measurement accuracy with time was estimated by taking the average, over all N = 350 time intervals, of the time-dependent ratios obtained for a specific time t.

### Basic experimental protocol

Prior to an experiment, colonies (a queen, 50–100 workers and brood) were starved (water was supplied *ad-lib*) for a period of 2–8 weeks. Even after eight weeks of starvation larvae and eggs are still present in the colony and there is no indication of massive worker death. On the day of the experiment, the colony (the queen and at least 95% of workers) is transferred to the experimental nest, which includes *ad-lib* water and then left undisturbed for at least four hours. Following this adjustment period, the colony is recorded for thirty minutes after which labeled food (in a glass tube plugged with cotton) is introduced to the nest yard.

Video titled ‘Colony food dissemination’, figs 3b, 4 and 5 (excluding the inset of Fig. 5a) relate to a sample experiment with the species *Camponotus sanctus* (two additional experiments with this species are presented in SI-9). In this experiment, the colony consisted of a queen, 50 workers, and no brood and was starved for 6 weeks prior to the measurement. A total of 1007 trophallaxis events were recorded by tracking the labeled food (a water-based solution of sucrose (40 g/l) and Rhodamine B (10 mg/ml)). The inset of Fig. 5a was taken from similar experiments that included a queen, 72 workers, and multiple brood items.

## Results

### Calibration and error estimation

**Figure 2:**
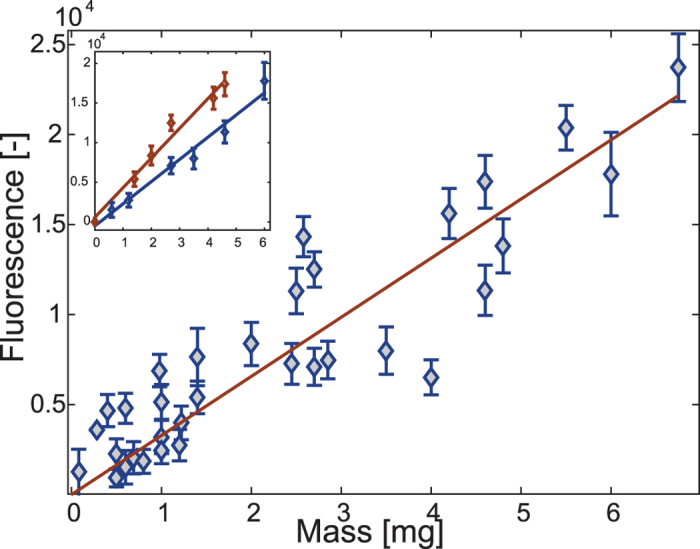
Fluorescence Calibration. Fluorescence signals (y-axis) are plotted vs. the ant’s food load as measured by direct weighing (x-axis). Measurement errors were estimated by applying two additional threshold values (for identifying a fluorescent pixel) in the image analysis that are 10% above or below the original one. Red line: linear fit with a slope of 3.3**∙**10^3^ [1/mg] , goodness of fit R^2^ = 0.87 (N = 7, mature minors of various sizes). **Inset**- two examples of single ant calibrations. Factors such as the variability between workers’ exoskeleton transparency induce differences in the slopes of the calibration curves (average goodness of fit : R^2^ = 0.98 ± 0.004, N = 7 individuals, x and y axes are the same as in the main figure).

We conducted a calibration experiment to estimate the accuracy of fluorescence imaging as a means of measuring the crop’s liquid content of an ant. This experiment relied on measuring ingested food both directly, by the increase in the ant’s total mass, and fluorescently (see Materials and Methods- Calibration of the fluorescence signal and SI-5). Pooling together calibration data from multiple individuals revealed a linear relation between the ingested food mass and the measured fluorescence (Fig. 2, goodness of fit: R^2^ = 0.87, 7 ants, 31 samples, average confidence interval of 0.74 ± 0.02 mg).

A considerable portion of the calibration error is due to inter-ant variation manifested as differences of up to 50% in the calibration slopes of individual ants (Fig. 2, inset). This may originate from variations in the cuticle transparencies of different ants. The calibration can therefore be further improved if it is separately performed for each individual (the average goodness of fit in this case is: R^2^ = 0.98 ± 0.004, N = 7 ants). Confidence intervals calculated from individual calibrations are reduced to an average of 0.3 ± 0.15 mg and sets the maximal accuracy that can be obtained using our system. The noise sources that contribute to this uncertainty include the volume of the opaque oesophagus and the variation in transparency along an ant’s cuticle. While the confidence interval is on the order of a small volume of crop liquid (*e.g*. if all food is in the oesaphagus, it will not produce a signal), it is reduced to 5% for a full crop (5.6 ± 0.3 mg, N = 6 ants).

The results presented in the previous paragraph imply that, in order to achieve maximal accuracy, the fluorescence signals from each ant must be scaled by her own transparency (which, for mature ants, remains constant over time^46^). Since performing dedicated calibrations for each ant prior to the experiment is impractical, the ant-specific scaling parameter must be estimated using the experimental data itself. Utilizing the fact that the total amount of food remains constant during trophallactic events, i.e. food donated by one ant must be received by the other, a transparency factor can be defined as:
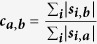


Where ***c***_***a,b***_ is the relative transparency factor of ant b with respect to ant a; *i* goes over all trophallactic events that occurred between these two ants; and ***s***_***i,v***_ is the absolute amount of liquids passed in the i-th trophallactic event, as calculated from measurements of ant ***v*** (***v*** = a or b).

Since the transparency factors are relative properties, they can be deduced by following trophallactic events during the course of an experiment (see Table 1). For example, the data in Table 1 was taken from a single experiment in which three workers were recorded inside a petri dish. At the beginning of the experiment, only one worker had labeled food in her crop. Each trophallaxis event gives us two measurements of the amount of liquid that was transferred: the decrease in the fluorescence of one ant and the increase in the measurement from the other. Calibrating the measurements associated to each ant by her relative transparency reduced the difference between these two measurements (the largest error was reduced from 1.05 to 0.21 mg). These corrected differences are on the order of the confidence interval of single ant measurements, implying that it is possible to practically eliminate the inter-ant variability.

The major sources of error discussed so far include the anatomical and transparency characteristics of ants. However, in an actual experiment another source of error comes into play. This error stems from the different postures that an ant assumes as she moves freely within the nest. Often an ant’s abdomen is obscured from the camera by her other body parts and this might result in errors that are on the scale of the measurement itself. Fortunately, these errors are transient and can be substantially reduced by considering only time intervals when the ant was not engaged in trophallaxis nor directly feeding. During such periods, the ant’s crop liquid content remains constant and therefore the maximal fluorescence measurement over all time points in this interval is the most reliable estimate of the crop liquid content for the entire interval (see Materials and Methods- Calibration of the fluorescence signal). For a 10-second period, this procedure yields estimates that are within 15% of data obtained using an ideal image, where the ant is maximally exposed to the camera. These errors are further reduced to an average of 10% for 30-second intervals. The average time that passes between trophallactic events is 240 ± 17 s (N = 1009), which is long enough to obtain a reliable measurement for the crop liquid content.

### Measuring food transfer during trophallaxis

**Figure 3:**
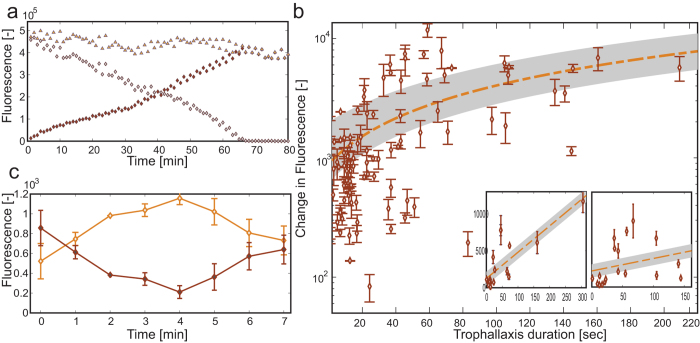
Trophallaxis measurements. (**a**) Fluorescence measurement of two ants during a single trophallactic event. Food passes in a unidirectional way from the donor to the receiver. Gray diamonds - donor crop contents, red diamonds - acceptor crop contents, yellow triangles - total fluorescence. (**b**) Amount of transferred food (the change in the individuals’ crop measurement before and after the event) vs. the trophallactic event duration. Data points depict the average change in fluorescence as measured from two workers that were engaged in the event, and the error represents the difference between the absolute value of each pair of measurements. The yellow line represents the best linear fit of the data, and the gray area marks the error bounds as estimated from the calibration. The data is widely spread, such that the correlation between the amount of transferred food and the event duration does not agree with a linear description. **Insets:** the amount of transferred food vs. the trophallactic event duration of two individuals sampled. These figures’ attributes are similar to Figure b, but the fluorescence measurements are taken from specific ants. (**c**) Back and forth trophallaxis between *Camponotus fellah workers*. Food initially passes from one worker (depicted by bright diamonds) to another (dark diamonds) but after five min. the direction changes. The error bars represent the difference between the total fluorescence in the measured image and the maximal total fluorescence measured during the entire trophallactic event.

Individual measurements of ant crop liquid content provide direct, quantitative assessments of the liquid flow during trophallactic events: as the crop liquid content of one ant depletes, the other fills up (see supplementary movies: ‘trophallaxis_sucrose solution’ and ‘trophallaxis_ labeled protein’. Species: *Camponotus sanctus*). During these events, the combined food level summed over both ants remains constant (Figs 1e and 3a). The amount of transferred food is set by the change in the fluorescence signals for each ant.

We used our method to reaffirm that observation of the mandible posture of two ants engaged in trophallaxis is a good predictor for the direction of food flow. We found that in 90% out of N = 44 events, two independent observers could identify distinct mandible postures that allow the determination of flow directionality (the donor’s mandibles are more widely gapped). Out of these ninety percent, the food flow, as determined by our novel method, agrees with that specified by the human observers in 88% of the cases. The other 12% exhibited no food flow.

We next inquired whether the statistics of trophallactic events can be used to determine whether, as was previously assumed^34^, simple event duration measurements could suffice in estimating trophallactic volumes. If event durations were linearly scaled with the transferred food volumes, then, given the linearity of the fluorescence signals (Fig. 2), one would expect a linear relation between total fluorescence change and event duration. However, this is not the case: even though longer event durations are correlated with larger volume transfers, the data is widely distributed, falling largely beyond the expected error (Fig. 3b).

An example showcasing a stark discrepancy between trophallaxis duration and transferred food volume is presented in Fig. 3c. During this single trophallactic interaction, the donor and the receiver identities switch; i.e. the direction of food flow reverses. Consequently, even though this interaction is long, the net amount of transferred food is small. Although bi-directional food transfer is rare (a few cases out of about a thousand trophallactic events) it emphasizes the requirement for direct measurements of crop liquid content and food exchange.

### Single ant dynamics

**Figure 4:**
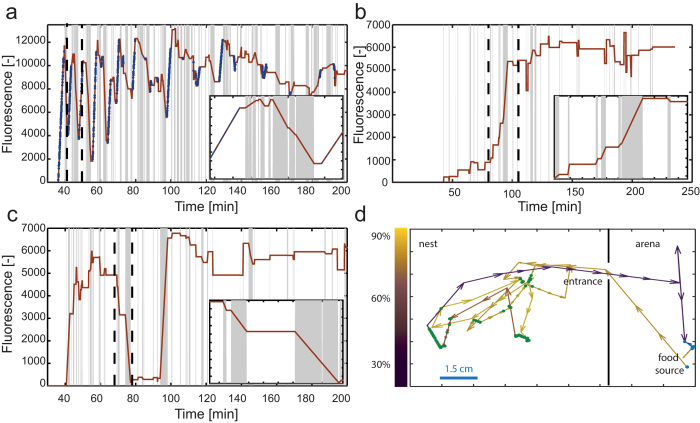
Individual timelines. **a- c** Food load timelines of individual ants: gray shading corresponds to trophallactic events, blue markers denote times at which ants were fed directly from the food source. **Insets**—enlarged images of the time intervals enclosed by the dashed vertical lines on the main panel. (**a**) A typical timeline of a foraging ant: following her first visit to the food source, this ant spends almost 30 minutes in back and forth trips between the food and the nest, each time loading food at the food source and unloading it in the nest. At later times, after most ants have received food, this behavior declines, and the forager’s crop contents do not change much. (**b**) A worker that had not left the nest, and therefore received its food load solely by trophallaxis. A series of small volume events is followed by a large event at time t = 95 min. (**c**) Massive food transfer by a non-forager: At time t = 70 min a cascade of trophallactic events begins, in which this non-forager ant gave away most of her its crop liquid conent, only to gain it back at later times. (**d**) A trajectory of the forager presented in panel a during a single trip from the food source to the nest and back (t = 43–45 min). The color of the arrows corresponds to the volume of crop contents as a fraction of the maximal measurement obtained from this ant. Green markers denote places in which trophallactic events took place, and blue markers denote direct feeding.

Our method produces a dataset that includes a timeline of the position of each individual ant, continued measurements of her crop liquid content, and a record of her interaction history. A typical timeline for a forager ant (*i.e*. an ant that has been to the food source at least once) includes a series of trips (13.7 ± 1.2, N = 10 foragers from two colonies) between the food source and the nest. In each trip the forager loads her crop at the food source and empties it in the nest by means of trophallaxis (Fig. 4a,d). On average, during each visit to the nest a forager transfers 49.8% ± 4.2% of her crop liquids at the time of entry to other ants (N = 30 visits during the first thirty min. after the first forager left the nest). As more ants in the colony are fed, forager ants exit the nest for their next foraging trip after unloading a smaller fraction of their crop contents (Fig. 4a). At even later times, this behavior ceases and the forager’s crop liquid content remains relatively constant. Most ants in the colony do not exit the nest to forage and are fed exclusively by trophallaxis. Non-foragers reveal a variety of food exchange behaviors. For example, some ants engage in a series of events during which they continuously increase their crop liquid content until it is full (Fig. 4b). Other non-foragers display a significantly different type of behavior: these ants both gain and distribute food (Fig. 4c). Additional data of single ant dynamics from a second colony is presented in SI-9 and shows similar behaviors.

### Collective measurements of Food dissemination

**Figure 5:**
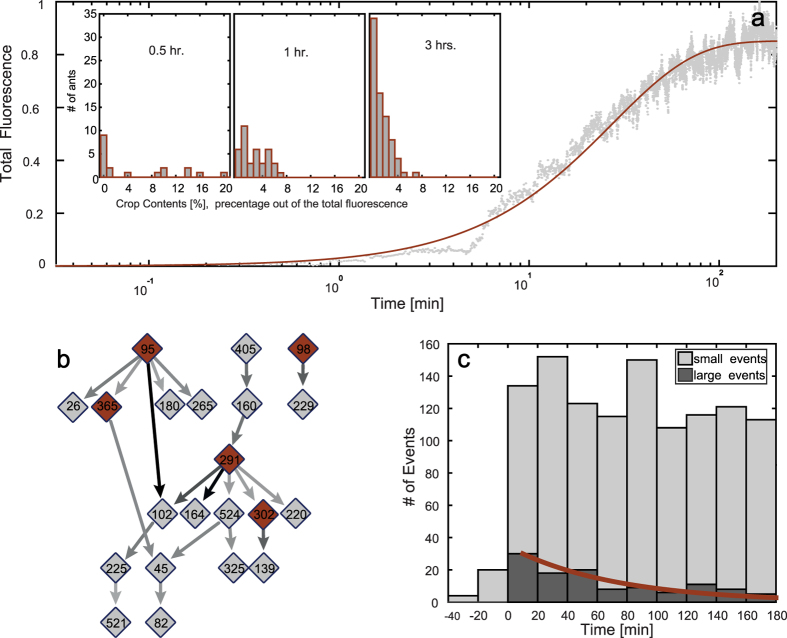
Collective aspects of trophallaxis. (**a**) Food accumulation: total colony intensity measurement vs. time Solid line: ***f*****(t) = 1 − *****e***^**−0.036*****t***^. **Insets:** food distribution of fed ants at times 0.5, 1 and 3 hrs after the first forager left the nest. These distributions correspond to the experimental data presented in SI-9 (Figure SI-4a). (**b**) Trophallactic network. The network (produced using Cytoscape software^58^) is constructed from those pairs that exhibited large net food transfer (greater than 1% of the total net food flow over all pairs). Highlighted nodes denote foragers, arrows indicate the direction and volume of the net flow in this connection. The darker the arrow the more (net) food that passed over this connection. (**c**) Time distribution of trophallactic events: number of large (dark bars) and small volume (bright bars) vs. time. Large volumes are defined as events in which the amount of transferred liquid is above the 90^th^ percentile. We therefore set this as a threshold to divide our data into small and large volume events. At t = 0 the first forager left the nest, bars before t = 0 include all trophallactic events. While the number of large volume events decays exponentially (solid line), the number of all trophallactic events remains high.

The global dynamics of food accumulation is in general agreement with previous measurements^39^, and can be approximated by ***F***** = *****F***_***max***_**(1** − ***e***^**−*****a**t*^), where F is the total fluorescence in the nest area , α is a fitted rate constant, and t is the time since the first forager returned to the nest (Fig. 5a and Figures SI-4a,b under SI-9). While the model describes continuous global phenomena by average parameters, in reality, trophallaxis events are discrete and the total fluorescence is equivalent to the amount of food ingested by the foragers directly from the food source. This deviation from the model is seen at early times when the total fluorescence level is low (Fig. 5a, ~4–5 min).

Our method provides additional information, including the distribution of food among the ants, which is non-uniform over space, time, and between individuals (Fig. 5a). This type of data can shed light on the collective dynamics leading to the steady state of long-term storage of food within the colony.

By their nature, trophallactic networks evolve constantly; connections between ants shift and change with time and the network is highly nested. However, if we focus on the most dominant connections, i.e. pairs with a net food flow (*i.e*. the total amount that one ant passed to the other minus the total amount that flowed in the other direction) that is larger than 1% of the total net flows between all pairs, a hierarchical structure containing no closed loops is revealed (see Fig. 5b and SI-8). In general, foragers tend to have an increased number of large volume connections with non-foragers (foragers have on average 31 ± 1 connections to other ants (N = 6) while workers have 21 ± 1 (N = 41)). Surprisingly, foragers not only disseminate food—they also receive a considerable amount of food from non-foragers. Therefore, foragers are not necessarily located in the roots of the hierarchical structure.

The distribution of event volumes (the amount of liquid passed in a single trophallactic event) falls off dramatically around the 90^th^ percentile. We therefore set this as a threshold to divide our data into small and large volume events. As soon as the first forager returns with food, the rate of small trophallactic events rises and does not decline for the entire duration of the experiment (Fig. 5c, data from an additional colony in SI-9). Differently, the number of large events decays within an hour following the onset of feeding and this corresponds to an increased number of fed ants (Fig. 5c). Small volume events are by far more frequent than large events and the total amount of food transferred in small events is twice the amount transferred in large events (during the 3 hour experiment). Finally, the high frequency of small events can be interpreted as a possible mechanism of information sharing regarding food availability, but it may also suggest that trophallaxis plays roles unrelated to nutrition, such as the maintenance of a gestalt colony odor^47^,^48^,^49^.

## Discussion

We present a method that enables precise bookkeeping of the process of trophallactic food dissemination in ant colonies and specify its accuracy and reliability. While previous methods perform reasonably well in determining the direction of flow, the method presented here provides a more accurate quantification of the trophallactic process and allows for measurements of the dynamics of food flow during individual trophallactic events. Furthermore, our preliminary results include a variety of qualitatively surprising results that were not observed before. Among these are (1) the switch in flow directionality during a trophallactic event, (2) foragers that not only unload food but also receive large amounts of it, (3) foragers that leave the nest after emptying only a small part of their crop liquid content, (4) non-foraging ants that still donate large amounts of food, and (5) the large total volume transferred by a large number of small interactions. We stress here that these observations are presented as a demonstration of the capabilities of the novel methodology. We leave study regarding the generality and significance of such observations to the general process of food dissemination to future work.

Our technique can provide insights into the low-level rules of trophallactic behavior. For example, the change in liquid food flow direction during a single trophallactic event may indicate that the donor and receiver roles are highly dynamic and continuously regulated. This example illustrates how detailed, quantitative measurements are crucial for achieving a precise phenomenological description of the interactions that make up this complex process. Once good descriptions of these low-level processes will be formulated, one will be able to draw connections between different scales of colony organization: the internal state of individuals (crop contents), pairwise trophallactic interactions, and the collective phenomenon of food dissemination.

In general, our experimental setup is unique in the sense that it provides not just a list of interactions—it also tracks the actual material transferred during these interactions. Such datasets are rare, especially in the case of biological systems, and thus hold promise for new perspectives on temporal networks and for elucidating the connection between their physical properties and functionality. These resulting insights may apply to more general questions such as the distribution of goods over dynamic networks^50^,^51^,^52^,^53^, the emergence of global patterns from microscopic interaction rules^3^,^54^,^55^, and regulation in decentralized control systems^56^,^57^.

Finally, an extension of our method can be used to study a more complex setting of multiple nutritional components. Labeling different nutrient sources with different colors, as well as adding adequate light sources and camera filters will enable simultaneous measurements of the flow of several nutritional components. Such measurements are especially interesting since different individuals in the colony have different dietary requirements. For example, larvae require a protein-rich diet, whereas workers ingest mainly carbohydrates^22^,^26^. Although it is known that essential nutrients are distributed differently in the colony^20^, multi-nutritional measurements would allow one to study how the rules ants use in individual trophallactic events vary to create multiple global patterns.

## Additional Information

**How to cite this article**: Greenwald, E. *et al*. Ant trophallactic networks: simultaneous measurement of interaction patterns and food dissemination. *Sci. Rep*. **5**, 12496; doi: 10.1038/srep12496 (2015).

## Supplementary Material

Supplementary Information

Supplementary Movie 1

Supplementary Movie 2

## Figures and Tables

**Table 1:** Trophallactic events between three ants, original and scaled data.

Donor name	Receiver name	Transferred food (based on donor fluorescence measurements) [mg]	Transferred food (based on receiver fluorescence measurements)[mg]	Error [mg]
A	B	2.78 ± 0.13	2.81 ± 0.09	0.035
		**2.78 ± 0.13**	**2.75 ± 0.09**	**0.02**
B	A	0.23 ± 0.19	0.19 ± 0.06	0.04
		**0.23 ± 0.19**	**0.19 ± 0.06**	**0.038**
B	C	1.44 ± 0.15	1.04 ± 0.06	0.42
		**1.41 ± 0.15**	**1.63 ± 0.09**	**0.22**
A	B	0.15 ± 0.04	0.13 ± 0.13	0.02
		**1.15 ± 0.04**	**0.13 ± 0.13**	**0.02**
B	C	1.26 ± 0.14	0.79 ± 0.13	0.47
		**1.23 ± 0.14**	**1.24 ± 0.21**	**0.002**
C	B	1.4 ± 0.12	2.45 ± 0.12	1.05
		**2.2 ± 0.12**	**2.4 ± 0.12**	**0.21**

In regular fonts, measurements of the transferred quantity calculated as the change in the fluorescence level of individual crops traslated to mg using the calibration line of Fig. 2 (y[mg] = 0.303 10^-3^ x). In bold, corresponding quantities scaled to ant A. The scaled data are the original data multiplied by the individual’s transparency factors. A transparency factor between two workers is defined as the ratio between their total transferred food measurements. Relative to ant A (whose transparency factor is set to 1), the transparency factors of ants B and C are 0.98 and 1.57, respectively. Since ant C did not have trophallaxis with ant A, her transparency factor is the multiplication of the transparency factor of ant C relative to ant B and the transparency factor of ant B with respect to ant A. The error is calculated as the difference between two independent measurements obtained by separately tracking the fluorescence signal changes from each of the two ants involved in the interaction.
