# Supplementary material for: Ant trophallactic networks: simultaneous measurement of interaction patterns and food dissemination
Source: Sci Rep. 2015 Jul 30;5:12496. doi: 10.1038/srep12496 (PMC4519732; doi:10.1038/srep12496)
Supplement: Supplementary Information [file srep12496-s1.pdf]

Supplementary Information

Ant trophallactic networks: simultaneous measurement of interaction patterns and food dissemination

Efrat Greenwald[1], Enrico Segre[2] and Ofer Feinerman[1,*]

1 Department of Physics of Complex Systems, Weizmann Institute of Science, Rehovot, Israel
2 Department of Physics Services Unit, Weizmann Institute of Science, Rehovot, Israel
* Correspondence to ofer.feinerman@weizmann.ac.il

Contents

1. Nutrient transfer via trophallaxis 3
2.Behavioral effect of the flashing light 4
3. Bleaching 5
4. Experimental arena size 6
5. Calibration 6
6. Associating ants with the corresponding fluorescent signal 7
7. Digestion 8
8. Network representation 8
9. Additional colony level data 9

1. Nutrient transfer via trophallaxis

To test whether ants may filter food transferred in trophallaxis such that fluorescent is passed while sugars are not we conducted a control experiment using sixty six workers. The ants were divided into the following groups: ants that fed directly from the food source (df), ants that fed directly from the food source and then acted as trophallaxis donors (do), ants that were fed solely by trophallaxis (st), and ants that were not fed at all(nf). The food source was a water based solution of Glucose (80gr/l) and Rhodamine B (8mg/l). To make sure that we were indeed use fed ants, the ants in the three fed groups (df, do, and st) were used only if they displayed a positive fluorescence signal.

At a second stage, the ants were dissected and their crops removed. The crops of all ants belonging to the same group were then pooled together into a single Eppendorf tube to increase the signal to noise ratio of the measurement. This entire procedure was repeated twice (df1: 8 ants and total sample weight: 3.7 mg, df2: 7 ants, 7.6 mg, do1: 9 ants, 6mg, do2: 10 antss, 9mg, st: 7 ants, 4.3 mg, st2: 7 workers 3.4 mg, nf1: 10 ants, 3.15 mg, nf2: 8 ants, 7.9 mg). To summarize we used 66 ants that were divided into 4 conditions. The number of repeats was chosen such that in each enough ants are pooled to obtain enough scrop volume such that it is sensed by the detector.

Inside the Eppendorf, we crushed the crops with a sharp needle to free any contained liquids. Each sample was diluted with 2 μl of purified water per mg of sample. The samples were then centrifuged for 10 min at 4°C, to rid the samples of any non-liquid substances. Each μl of the liquid samples was then further diluted with 40 μl of purified water, vortexed and then measured for Glucose using a Blood Glucose Monitoring System ('FreeStyle-Optimum', Abbott).

The results show that the ants that were fed solely by trophallaxis had similar glucose concentration as the directly fed ones, and much above the concentration level of the starved ants (fig. SI-1a). The values in Fig. SI-1a are the average of two measurements, and the error is the difference between the measurements values and the mean. In the case of the ants that were not fed at all (nf), one measurement was below the detection level of the monitor (which is 20 mg/dl). In this case the presented value is the higher level measurement (24 ±10mg/dl). Calibration curve of known glucose concentrations in a water based Rhodamine-B solution is shown in figure SI-1b showing that Rhodamine B has no impact on the glucose concentration measurement of the monitor.

Another indication of the passage of nutrients via trophallaxis is demonstrated in the case of the protein Adenylate Kinase (see movie- trophallaxis with labeled protein) in which the fluorescent marker is bound to the nutrient itself (fluorescent marker: ATTO590 at position 169, solution concentration: 5μM in 50% glycerol). In this case of the protein Adenylate Kinase, fluorescent measurements are completely equivalent to the protein content.

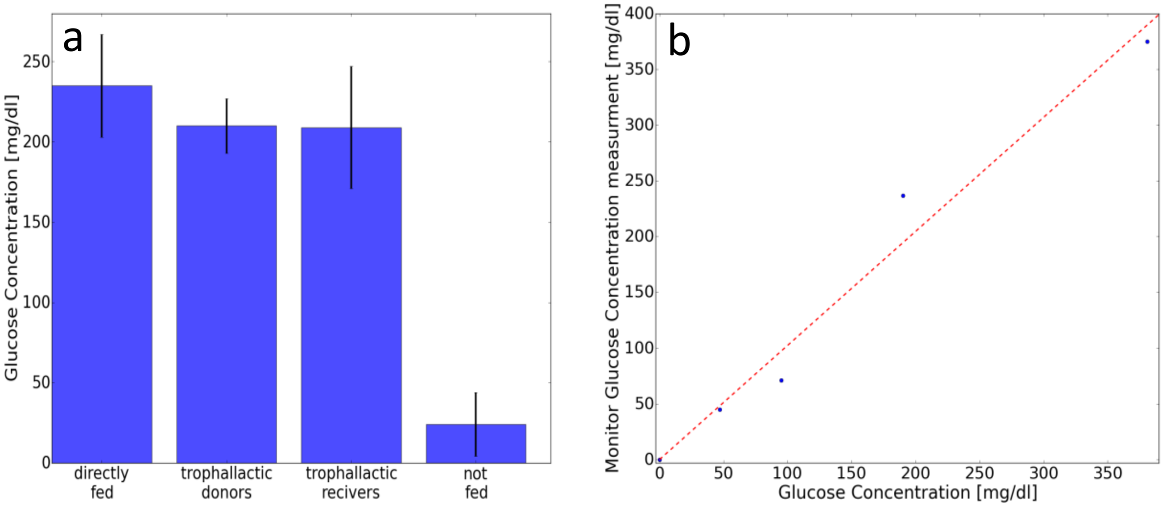

Figure SI-1. Glucose concentration measurements. **(a)** Crop liquids glucose concentration of four worker groups: directly fed, trophallaxis donors that were directly fed, ants that were fed solely by trophallaxis, and ants that were not fed at all. The results show that the ants that were fed solely by trophallaxis had similar glucose concentration as those that fed directly, and much above the concentration level of the starved ants. This indicates that glucose is indeed transferred with the Rhodamine B labeled solution. **(b)** Calibration data of the glucose monitor with water based glucose Rhodamine B solutions. The linear calibration curve ($y = 1.023\ x$, $r^2=0.97$) indicates that the presence Rhodamine B in the solution had no impact on the glucose concentration measurements.

2. Behavioral effect of the flashing light

We recorded more than 100 ants in three consecutive periods of thirty min length. The first two period ants were exposed only to IR illumination, and in the third period the pulsed light was operated (arena size: 160 cm^2, acquisition frame rate: 1 Hz, pulsed light rate 1Hz). We repeated this experiment on two consecutive days and measured the mean velocity and the mean path of the ants. The results presented in SI-table-1 show that the effect of the flashing lights is on the order of,

or even smaller than, the natural variation of the ants' behavior under constant conditions.

	# of ants	Mean path (1st dark period) [m]	Mean path (2nd dark period) [m]	Mean path (pulsed light) [m]	Mean velocity (1st dark period) [mm/s]	Mean velocity (2nd dark period) [mm/s]	Mean velocity (pulsed light) [mm/s]
Colony 1 (trail 1)	79	0.8±0.44	0.75±0.4	0.75±0.3	0.46±0.05	0.45±0.05	0.45±0.046
Colony 1 (trial 2)	30	0.19±0.25	0.24±0.74	0.37±0.64	0.1±0.05	0.13±0.07	0.2±0.08
Colony 2 (trial 1)	36	0.45±0.5	0.47±0.43	0.54±0.5	0.25±0.05	0.27±0.06	0.3±0.05
Colony 2 (trial 2)	69	0.49±0.4	0.4±0.3	0.46±0.28	0.27±0.04	0.22±0.03	0.25 ±0.03

SI-Table 1. The effect of the flashing light on ants . The mean path and the mean velocity were measured in three consecutive times: Two dark periods followed by flashing light period.

3. Bleaching

Bleaching was tested by analyzing the decay in fluorescence of solution drops laid on the glass floor (ten millisecond light pulses at a rate of 1Hz). The fluorescence measurement (fluorescence measurement per area) declined by 1% in two hours and by 8% within 5 hours (Figure SI-2).

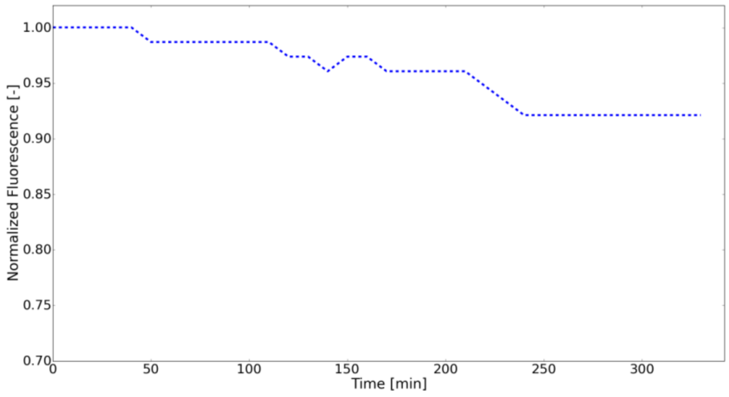

Figure SI-2. Bleaching of the Rhodamine B solution. The fluorescence measurement (fluorescence measurement per area) declined by 1% in two hours and by 8% within 5 hours. Therefore bleaching is a minor error source during the course of the main dissemination process, which takes place in around one hour.

4. Experimental arena size

Our camera resolution is 25 MP. Dividing 25 MP by a tag's pixel area (28x28) and multiplying by the tag's physical area (1 mm^2) we find that the permitted size of the experimental arena is 318 cm^2.

5. Calibration

Calculation of the confidence intervals from the calibration data. A linear fit to the measurements ({(x_i,y_i}, i=1..31) is taken as a calibration curve, y=ax+b, that we can use to translate the measured unit y (in our case fluorescence) into the desired unit x (mg in our case). The approximation used by Miller (referred to in the text) is a method that uses the deviations of the measured points from the fitted line in order to calculate confidence bounds on the mass of the liquid given its measured fluorescence. According to this approximation, for a specific fluorescent measurement y_0, the confidence interval, s_{x_0} indicating how well the value of x as calculated from y_0=ax+b estimates the actual mass x_0 is:

$$s_{x_0} = \frac{s}{a}\sqrt{\frac{1}{m} + \frac{1}{n} + \frac{(y_0 - \bar{y})^2}{a^2 \sum (x_i - \bar{x})^2}}$$

Where:

a - is the slope of the linear calibration curve, n – is the number of points in the calibration curve, m – the number of replicates of the measurement and s – the variance of the calibration curve: $s^2 = \frac{\sum (y_i - \bar{y})^2}{n-2}$

Since we wanted to estimate the accuracy of our measurements, we calculated the confidence interval for each of the 31 points in the calibration curve separately. This was done by excluding the point from the dataset, performing the fit and calculating the confidence interval as if the point was the measurement y_0 .

To get measurement error we multiplied the confidence interval, s_{x_0}, by t value of two tailed t distribution with 28 (=31-1-2) samples and 95% confidence.

6. Associating ants with the corresponding fluorescent signal

Our method utilizes a double camera system and this leads to discrepancies in both space and time (due to non-perfect alignment and synchronization). However, we calibrate our system such that these discrepancies are minimized (to be well under the length scale of an ant). We then use a post-processing step in which we associate the tags of ants to the blobs for all cases in which we can be sure that there is a match (discarding a small fraction of the data). This step ensures that ants are associated with the correct blobs even if these seem slightly removed in the images themselves. The two main reasons for non-perfect alignment between the light image of an ant's gaster and the fluorescent image of her crop contents are:

1. Space. The axes of the two cameras are not exactly parallel to each other, the cameras have different lenses, and the areas they image do not precisely match. Although these inconsistencies are minimized by careful alignment of the system some mismatch must always remain. This mismatch is handled by post-processing in which the coordinates of one image are transformed so that they are overlaid over those of the other. The transformation does not result perfect overlay in 100% of the images' area, but, nevertheless a very close one – such that the difference between an ant's gaster and the fluorescent signal that it elicits does not go over 0.5 mm or 0.25% ant lengths.

2. Time: The fluorescent camera serves as a master camera: it triggers both the upper camera and the fluorescent illumination to minimize time differences between the two images. However, such differences are cannot be completely removed because the differences in camera reaction and exposure times. This causes discrepancies that are on the order of 50ms. Over this time scale fast ants (typically walking outside the nest) moving at 10cm/sec can pass about 5 mm and leads to a discrepancy between the ant and her fluorescent signal.

Careful calibration of the experimental system allows us to minimize these small mismatches. We then set to associate tags to blobs offline. This process uses the Munkres algorithm to associate blobs to tags using the distance between them. Since errors are typically smaller than an ant's length this gives accurate results. While making these associations, we include an upper bound distance and do not associate tags and blobs that are further away. This increases the accuracy of association at

the price of deleting some relevant frames from our data-set (this is not problematic since the movie includes a large number of frames). Finally, we generate a movie which combines the data-streams of the two cameras and depicts the automatically generated associations between barcodes and blobs. We use this movie to double-check the associating algorithm performance. Any errors (typically less than 1%) may be manually corrected.

7. Digestion

The timescale of digestion was tested by measuring fluorescence levels of fed workers while being isolated (N=9 ants, species: *Camponotus sanctus,* food source*:* a water-based solution of glucose (80 gr/l) and Rhodamine B (0.08 mg/ml)). By 15 hours (900 minutes) the average signal was reduced by 10% (See Fig. SI-3). This value is not much different from the decay that could be expected from bleaching alone. This time scale is also consistence with previous studies (reference 40).

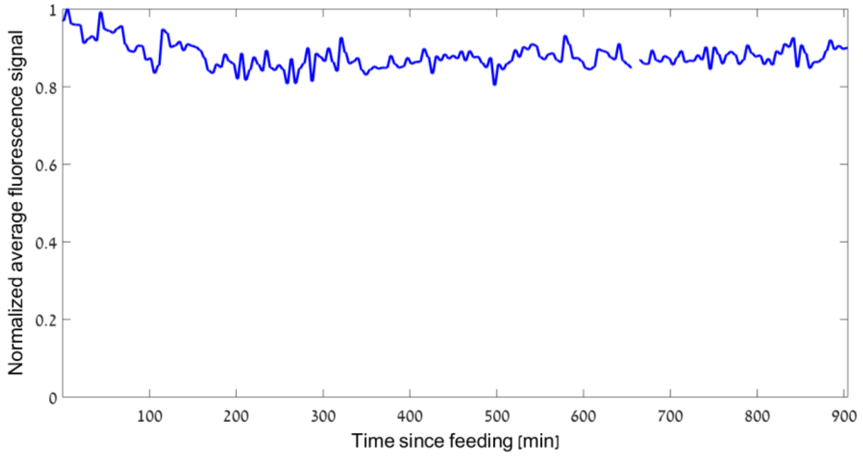

Figure SI-3. Fluorescence measurement of isolated workers of *Cmponotus sanctus* after being fed with fluorescently labeled glucose solution, averaged over 9 ants. By 15 hours (900 minutes) the average signal was reduced to 90% of the initial fluorescence level.

8. Network representation

The trophallaxis network is a dynamic network. To represent it we chose a time window (3 hours) and considered all interactions within it. For each pair of ants both the absolute and the net food flow transferred between them was separately calculated over the course of the first three hours of the experiment.

The absolute flow was calculated as the sum of all absolute values of fluorescence measurements of liquid transfer, and the net as the sum of measured food flows from one ant to the other minus the sum of the measurements in the opposite direction. The edges presented in figure 5b are those whose net flow represents more than 1% of the sum of all net flows over all pairs. The direction of the arrow (in figure 5b) signifies the direction of positive net flow and the shade of the arrow represents the amount of net food flow. The hierarchical network representation option in Cytoscape (see reference 58 of the main text) reveals that, in this case, the links of large flow constitute a pure hierarchical network.

9. Additional experimental data

Experimental data for experiments as described in the basic experimental protocol (see Materials and Methods-Basic experimental protocol of the Main manuscript). Colony 1 (Fig. SI-4a) included a queen, 72 workers, and broods. Level of starvation 7 weeks. Colony 2 (Fig. SI-4b) included a queen, 91 workers, and broods. Level of starvation 8 weeks. In both experiments food source was a water-based solution of sucrose (80 gr/l) and Rhodamine B (0.08 gr/l). Data is presented for:

1) Food accumulation within *Camponotus sanctus* colonies, (Colonies 1-2).
2) Single ant dynamics, similar to that presented in figure-4 of the main text (Colony 2).
3) Time-line of the occurrence of events grouped by either small or large size (Colony 2).

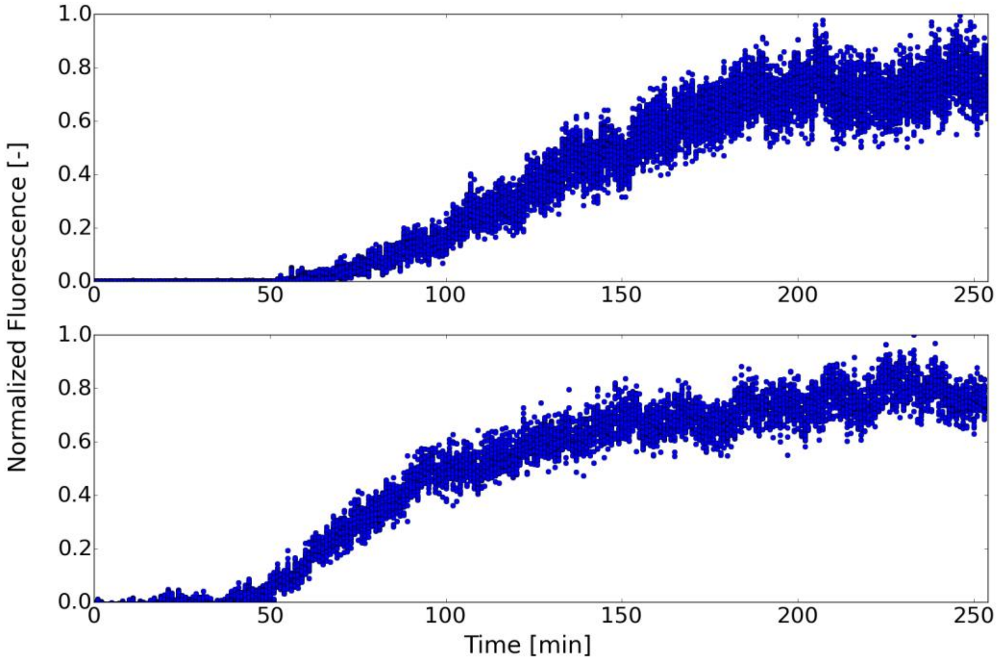

Figure SI-4. Food accumulation in *Camponotus sanctus* colonies: Normalized total Fluorescence vs. time. (Fig. (a) Colony 1: including a queen, 72 workers, and broods starved for 7 weeks (b) Colony 2: including included a queen, 91 workers, and broods starved for 8 weeks. In both cases, food was introduced at t=0 and the first forager entered the colony at t=50, 35 min ,(colony 1, 2 respectively).

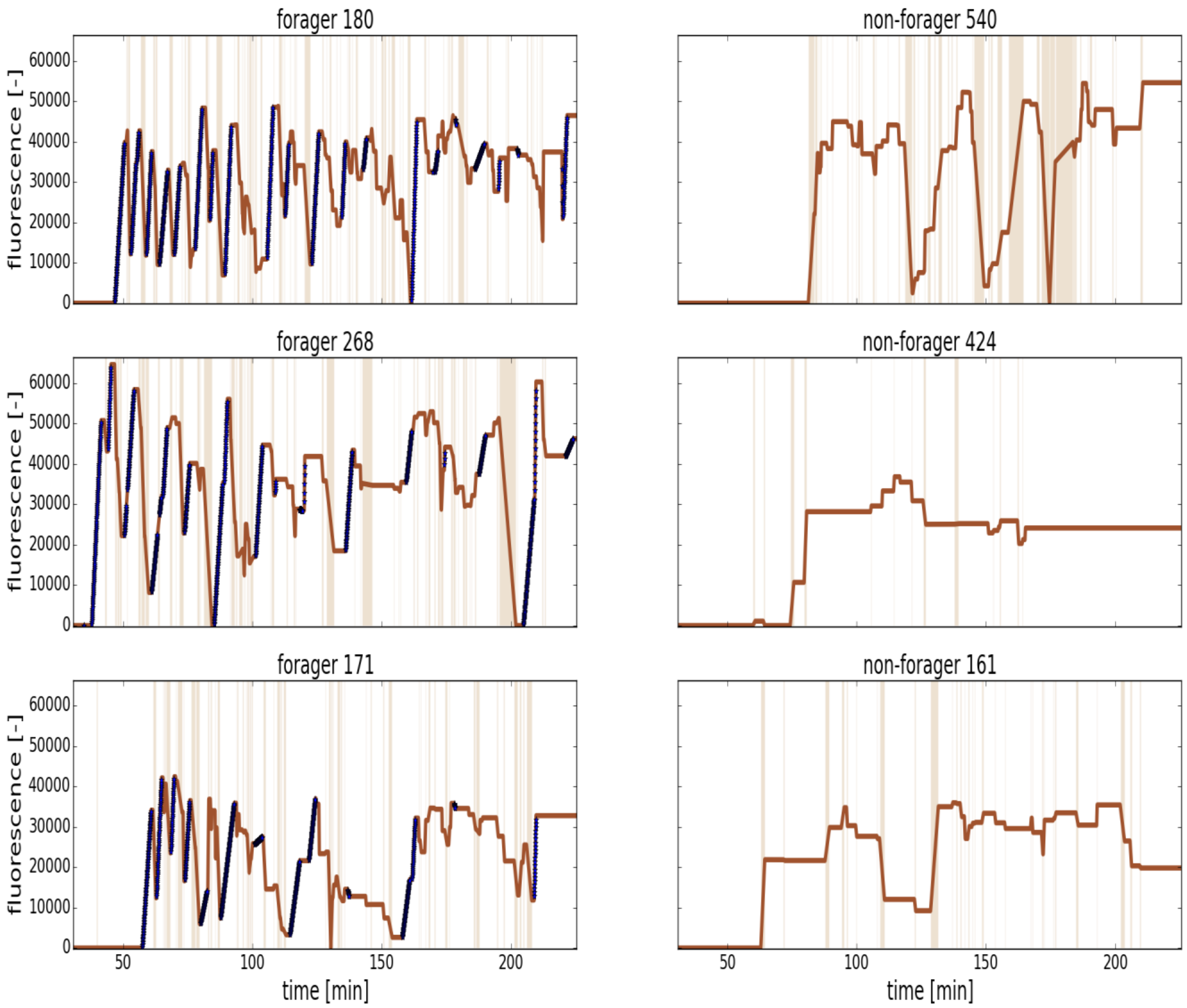

Figure SI-5. Time lines of the crop liquid contents of sample ants: gray shading corresponds to trophallactic events, blue markers denote times at which ants fed directly at the food source. Foragers go back and forth between the nest and the food source. All ants are of colony 2.

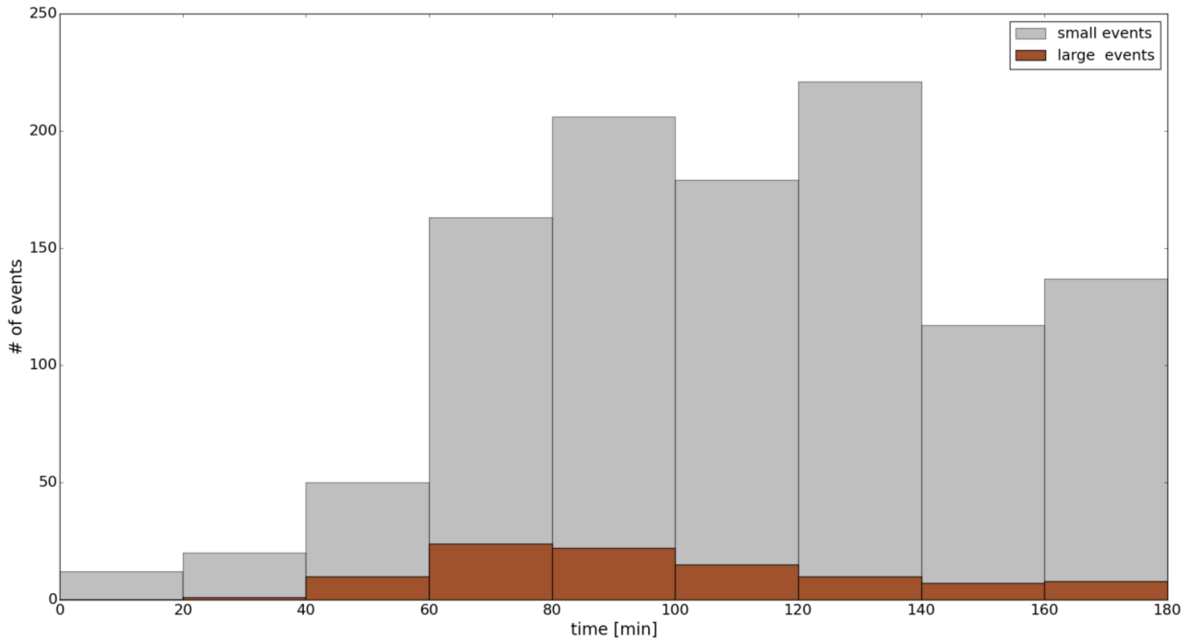

Figure SI 6. Time distribution of trophallactic events: number of large (brown bars) and small (gray bars) volume events vs. time, colony 2. At t~35 min the first forager left the nest. Bars prior to t=20 include all trophallactic events. Treshold value for large volume event was arbirarily set to be the 90th p ercentile.
